# Utilizing Crowdsourced Data for Studies of Cycling and Air Pollution Exposure: A Case Study Using Strava Data

**DOI:** 10.3390/ijerph14030274

**Published:** 2017-03-08

**Authors:** Yeran Sun, Amin Mobasheri

**Affiliations:** 1 Urban Big Data Centre, School of Social and Political Sciences, University of Glasgow, Glasgow G12 8RZ, UK; 2 GIScience Research Group, Institute of Geography, Heidelberg University, D-69120 Heidelberg, Germany; a.mobasheri@uni-heidelberg.de

**Keywords:** crowdsourced data, Strava Metro, cycling purpose, air pollution exposure, particulate matter

## Abstract

With the development of information and communications technology, user-generated content and crowdsourced data are playing a large role in studies of transport and public health. Recently, Strava, a popular website and mobile app dedicated to tracking athletic activity (cycling and running), began offering a data service called Strava Metro, designed to help transportation researchers and urban planners to improve infrastructure for cyclists and pedestrians. Strava Metro data has the potential to promote studies of cycling and health by indicating where commuting and non-commuting cycling activities are at a large spatial scale (street level and intersection level). The assessment of spatially varying effects of air pollution during active travel (cycling or walking) might benefit from Strava Metro data, as a variation in air pollution levels within a city would be expected. In this paper, to explore the potential of Strava Metro data in research of active travel and health, we investigate spatial patterns of non-commuting cycling activities and associations between cycling purpose (commuting and non-commuting) and air pollution exposure at a large scale. Additionally, we attempt to estimate the number of non-commuting cycling trips according to environmental characteristics that may help identify cycling behavior. Researchers who are undertaking studies relating to cycling purpose could benefit from this approach in their use of cycling trip data sets that lack trip purpose. We use the Strava Metro Nodes data from Glasgow, United Kingdom in an empirical study. Empirical results reveal some findings that (1) when compared with commuting cycling activities, non-commuting cycling activities are more likely to be located in outskirts of the city; (2) spatially speaking, cyclists riding for recreation and other purposes are more likely to be exposed to relatively low levels of air pollution than cyclists riding for commuting; and (3) the method for estimating of the number of non-commuting cycling activities works well in this study. The results highlight: (1) a need for policymakers to consider how to improve cycling infrastructure and road safety in outskirts of cities; and (2) a possible way of estimating the number of non-commuting cycling activities when the trip purpose of cycling data is unknown.

## 1. Introduction

Over the last two decades, researchers have provided much evidence of the benefits of cycling as a health-enhancing physical activity [1,2,3,4,5,6,7]. Recently, volunteered geographic information (VGI), user-generated content (UGC) and crowdsourced data are becoming promising data sources for transport and health research [8,9]. Traditional methods of collecting cycling data, including manual counts, stated preference surveys [10,11] and annual average daily bicycle (AADB) volumes [12], are expensive and time-consuming. Each of these methods has its advantages, but each is almost impossible to accomplish over a broad area simultaneously, which is why crowdsourced methods are gaining interest in planning [9]. Through the expansion of Global Positioning Systems (GPS) new methods for collecting detailed cycling route information have emerged [13]. GPS-enabled mobile devices, such as smartphones, allow individuals to track and map their cycling routes [13,14,15,16]. More recently, crowdsourced cycling data are used to analyze cycling behavior [13,17] and make associations between cycling and health [9]. Strava is a popular website used to track users’ cycling and running activity via GPS-enabled devices, such as smart phones and smart watches. Millions of people upload their rides and runs to Strava every week via their smartphones or other GPS devices [18]. Strava launched a data service called Strava Metro that offers aggregated data sets after anonymizing and aggregating individual’s GPS traces. In earlier studies that use traditional data collection methods, research on the role of cycling for health through physical activity has been limited by the lack of information on where bicyclists ride [9]. With a high spatial resolution, Strava Metro data is able to provide new opportunities for research into active travel, sustainable travel and public health. This could benefit studies of cycling behavior and public health, and further help policymakers in urban planning, especially designing urban infrastructures aiming to make urban residents healthier and cities more sustainable. For instance, knowing where people like to cycle could help policymakers to improve cycling infrastructure more effectively (e.g., availability of cycle parking in areas of high demand) and promote road safety by giving priority to roads where there are more cycling trips. In several recent studies, Strava data has been used to map ridership over a city [13], evaluate the impact of bicycle infrastructure on cycling behavior [17], and investigate impacts of residential and employment density, land use diversity, cycling facilities and terrain on cycling behavior [9].

The impact of outdoor and traffic-related air pollution on health is an important issue in transport and health [19,20,21,22,23,24,25,26,27,28]. Typically, impacts of active travel (cycling and walking) and inactive travel (traveling by car, bus or train) on health are compared [22,23,24,25,26,27]. Although earlier studies offer much evidence on the health benefits of cycling or walking due to increased physical activity [1,2,3,4,5,6,7], some other studies reveal cycling also carries some potential health risks, including air pollution, accidents and noise [29,30,31]. One of the most important risks is from poor air quality [29,30]. Exposure to air pollution is harmful to human health [32,33,34,35,36,37,38,39,40,41,42,43,44] and more than 80% of people living in urban areas that monitor air pollution are exposed to air quality levels that exceed World Health Organization (WHO) safe limits [32]. Cyclists riding in urban areas are therefore likely to be exposed to high levels of air pollution. Recent studies use health impact modelling (HIM) to estimate the health benefits and risks of active travel (cycling, walking), and reveal that the total benefits of active travel outweighed the risks [28,45,46]. Particularly, a very recent study reveals that benefits of active travel outweighed the harm caused by air pollution in all but the most extreme air pollution concentrations [28]. It is also becoming widely accepted that increasing cycling time tends to increase health improvements. However many such studies assess cyclists’ exposure to air pollution based on city-level air pollution values when in fact, air pollution levels vary spatially over a city. Relating cycling activities to air pollution at a larger scale (e.g., street-level) could promote assessment of air pollution exposure when it is known where and when cyclists ride in a city. Ideally, urban planners and policymakers could use this knowledge of where cyclists ride to devise cycling and walking routes that minimize the risks faced by active commuters, and to decrease volume of cyclists riding in the environments that are associated with the highest exposures [29,47].

Moreover, Strava Metro data also indicate cycling purpose (commuting or non-commuting) of cycling activities. Researchers might make use of this in studies of cycling purpose and health. In this paper, we explore the potential of Strava Metro in research of active travel and health by using the data to investigate spatial patterns of non-commuting cycling activities and associations between cycling purpose (commuting and non-commuting) and air pollution exposure at a large scale. Additionally, as some cycling trip data sets (e.g., crowdsourced GPS trajectories or bike-sharing origin-destination trips) lack trip purpose (commuting or non-commuting), we can’t directly relate cycling purpose to air pollution exposure when utilizing those data sets. However, we might estimate the number of non-commuting cycling trips based on the number of all-purpose trips and environmental characteristics that affect cycling behavior. In this paper, we try to estimate the number of non-commuting cycling trips based on the number of trips for all purposes and environmental characteristics, as the estimation model could be validated by the Strava Metro data. If this method of estimating the number of non-commuting cycling trips is shown to be good, we may then use it to estimate the number of other non-commuting cycling trip datasets where trip purpose is unknown.

In this paper, we use the Strava Metro data in Glasgow, UK to carry out an empirical analysis. Firstly, in order to explore spatial patterns of non-commuting cycling activities, we investigate where non-commuting cycling activities are more likely to be than commuting cycling activities by identifying clusters where there are high rates of non-commuting cycling activities. Afterward, to associate cycling purpose with air pollution exposure at a large scale (i.e., the street intersection level), we investigate whether cyclists riding for recreation and other purposes (excluding commuting) are more likely to be exposed to relatively low levels of air pollution than cyclists riding for commuting. Note that levels of air pollution also might also vary over time in the study area. We focus on spatial variations of air pollution levels, not spatio-temporal variations of air pollution levels as temporal resolution of the air pollution data is one year. In this study, we focus on the difference in air pollution exposure during cycling, not the difference in health effects of cycling. Additionally, we strive to improve the estimation of the number of non-commuting cycling activities by using different regression methods (linear and non-linear methods) as the estimation models.

## 2. Materials and Methods

In this section, the methods used for spatial analysis of non-commuting cycling activities and air pollution are presented. Section 2.1 introduces the data and study area, Section 2.2 explores spatial patterns of non-commuting cycling activities and associations between cycling purpose and air pollution exposure, and Section 2.3 presents how we estimate the number of non-commuting activities according to total cycling activities and locational characteristics.

### 2.1. Data and Study Area

#### 2.1.1. Strava Metro Data

Strava (San Francisco, CA, USA) is a popular online social network for cyclists and runners with a user base larger than other similar sites like MapMyRide (Under Armoour, Baltimore, MD, USA), MapMyRun (Under Armour, Baltimore, MD, USA) or RideWithGPS (Ride with GPS, Portland, OR, USA). Strava consists of a mobile app and a website, allowing users can track their rides, runs, walks and hikes on a smartphone or another GPS device. The Strava app records distance, time, average speed and route (GPS trajectory) of each activity. Users can also add textual information like titles and tags to describe their trips. The ‘commute’ flag indicates walking or riding journeys to or from work. Users can upload their GPS-tracked activities recorded by their Strava apps to the Strava’s website (https://www.strava.com). Strava’s database comprises nearly a trillion GPS points globally and is growing by over 8 million activities every week [48].

Strava Metro is a suite of data services that enables cutting-edge views into cycling and pedestrian (running, walking, hiking) patterns [49]. Aiming to produce state-of-the-art spatial data products and services to make cycling, running, and walking in cities better, Metro anonymizes and aggregates activity data from Strava’s millions of users and then collaborates with departments of transportation and city planning groups to improve infrastructure for bicyclists and pedestrians [49]. To acquire the number of commuting activities, Metro first count ‘commute’ flags. As some users tend to not select the ‘commute’ flag, Metro further uses two ways to detect commuting activities from activities where ‘commute’ is not flagged: (1) activities with keywords in the titles such as “To Work” and “Commute To”; and (2) starting and ending points more than 1 km apart within a distance and time threshold (This can be user defined but we find 30 miles and 90 min to be a good top threshold) [48].

More than half of the Strava Metro’s dataset for dense metropolitan areas corresponds to commuting trips [49]. Some studies suggest crowdsourced data may be a good proxy for estimating daily, categorical cycling volumes by comparing cyclist counts between Strava data and manual count data in count stations [13,50]. For example, the Oregon Department of Transportation (ODOT) found the month-to-month correlation on the Hawthorne Bridge between total number of bicycles counted with a bike counter and total number of Strava bicycles trips over a one-year period was an adjusted R-Squared 0.91 [50]. Although crowdsourced cyclists represent a small portion of all cyclists, comparison with manual counts revealed a linear relationship between crowdsourced cyclists and total ridership in Victoria, Canada [13]. Due to an ability of tracking cycling activities at a high level of spatial granularity and a fairly high representation of the population of cyclists, Strava Metro data seem to have a high potential for studies of active travel and health in urban areas.

**Figure 1:**
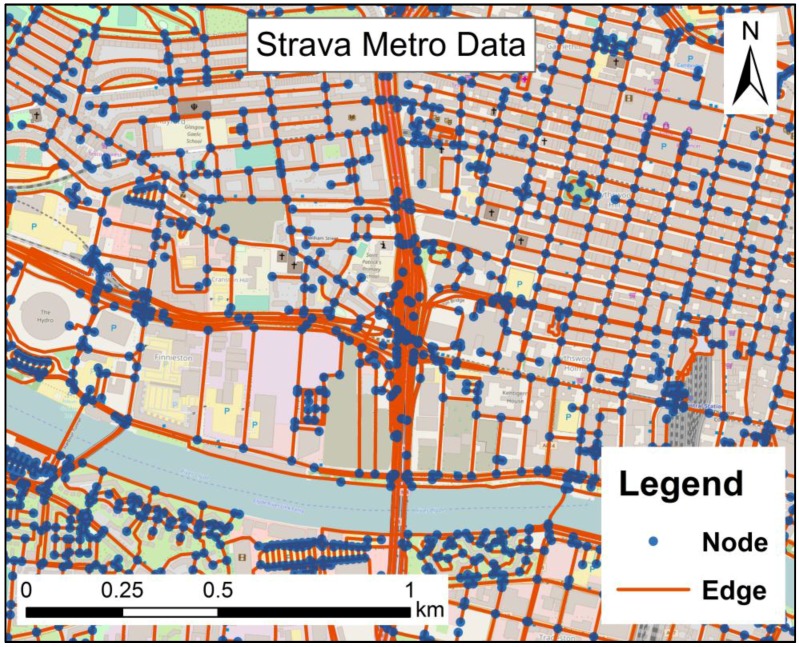
Nodes and edges of Strava Metro data (Basemap: OpenStreetMap, licensed under the Open Database License (ODbL)).

The Urban Big Data Centre, UK offers a Strava Metro dataset to researchers [51]. This dataset records 287,833 cycling activities (174,758 commuting activities and 113,075 non-commuting activities) contributed by 13,684 users (11,216 males, 1698 females and 770 blank-gender users) within the Glasgow Clyde Valley Planning area (including Glasgow City and seven council areas) in 2015. This dataset contains three sub sets in three formats: Streets, Origin-Destination and Nodes (see [49]). In this study, we select the sub set Nodes. A node represents an intersection of street and an edge represents a street (see Figure 1). The street network is extracted from OpenStreetMap (OpenStreetMap Foundation, West Midlands, UK). Attributes of a node includes count of all-purpose cycling activities at the intersection in 2015 and counts of commuting activities at the intersection (node) in 2015. Note that Strava Metro uses the number of all-purpose cycling trips (regardless of unique riders) that meet at the intersection to represent count of all-purpose cycling activities at the intersection, and uses the number of commuting cycling trips (regardless of unique riders) that meet at the intersection to represent count of commuting cycling activities at the intersection. We can infer that count of the non-commuting activities at the intersection (node) equals the difference between count of all-purpose cycling activities and count of commuting activities at the intersection (node). Additionally, the dataset contains a file that offers demographics of the bike trips, including average distance (24 km), median distance (15 km), average time (1.34 h), and median time (0.77 h).

**Figure 2:**
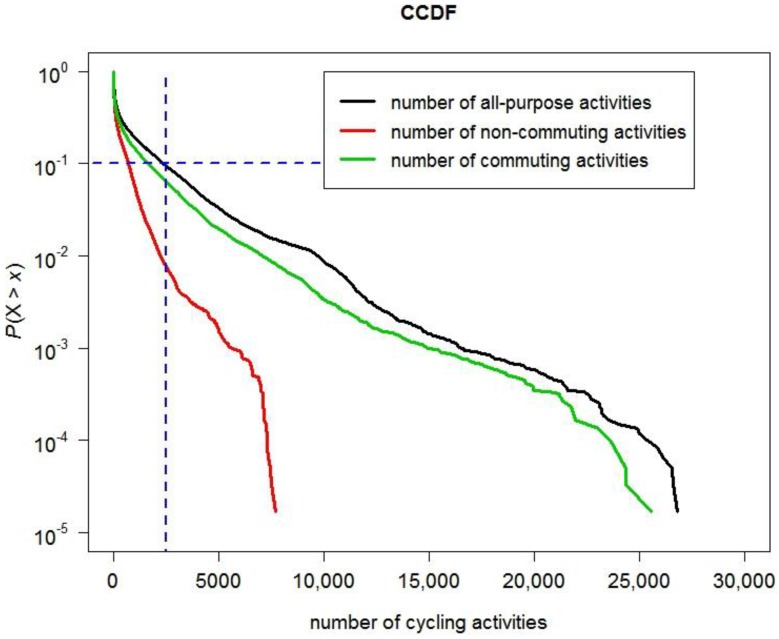
Cumulative distributions of numbers of all-purpose cycling activities, non-commuting cycling activities and commuting cycling activities in the log-linear plot.

Additionally, we look at the distributions of numbers of all-purpose cycling activities, inferred non-commuting cycling activities and commuting cycling activities in a node. Figure 2 shows cumulative distributions of numbers of all-purpose cycling activities, non-commuting cycling activities and commuting cycling activities in a node by using the complementary cumulative distribution function (CCDF). The CCDF of a variable X at *x* represents probability that X takes a value more than *x*, i.e., *P*(X > *x*). For instance, CCDF of the number of all-purpose cycling activities at 2500, i.e., *P*(X > 2500), equals to 0.1. This means that 10% of nodes have a relatively large number of all-purpose cycling activities (e.g., more than 2500); whilst the other 90% of nodes have a relatively small number of all-purpose cycling activities (e.g., less than or equal to 2500). Intuitively, the cumulative distributions of numbers of all-purpose cycling activities, non-commuting cycling activities and commuting cycling activities all seem to approximately follow an exponential law as they look like straight lines in the log-linear plot (see Figure 2). Note the log-linear plot is a semi-log plot with a logarithmic scale on the *y*-axis and a linear scale on the *x*-axis.

**Figure 3:**
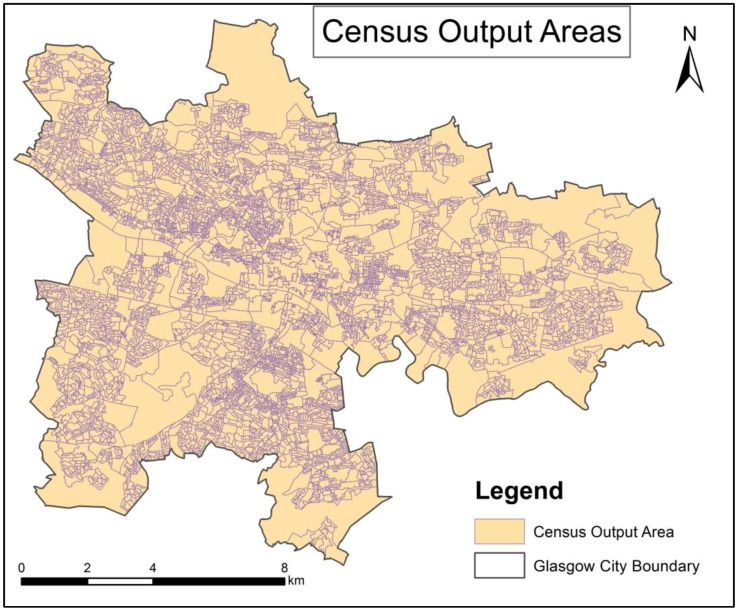
Census output areas in Glasgow (source: DATA.GOV.UK [52]).

Within the administrative boundaries of Glasgow, there are 59,718 nodes. Among those nodes, a few of nodes seem to have null or incorrect records. For instance, in some nodes count of commuting cycling activities is larger than count of all-purpose cycling activities; while in some other nodes counts of all-purpose cycling activities is zero. After removing these noise nodes, 50,057 nodes are kept as the data set. In the spatial analysis, we first need to aggregate nodes to areas as we want to identify clusters consisting of contiguous areas. In this study, the area unit is census output area. There are 5486 census output areas in Glasgow (see Figure 3). The census output areas data is downloaded from DATA.GOV.UK [52].

#### 2.1.2. Air Pollution Data

**Figure 4:**
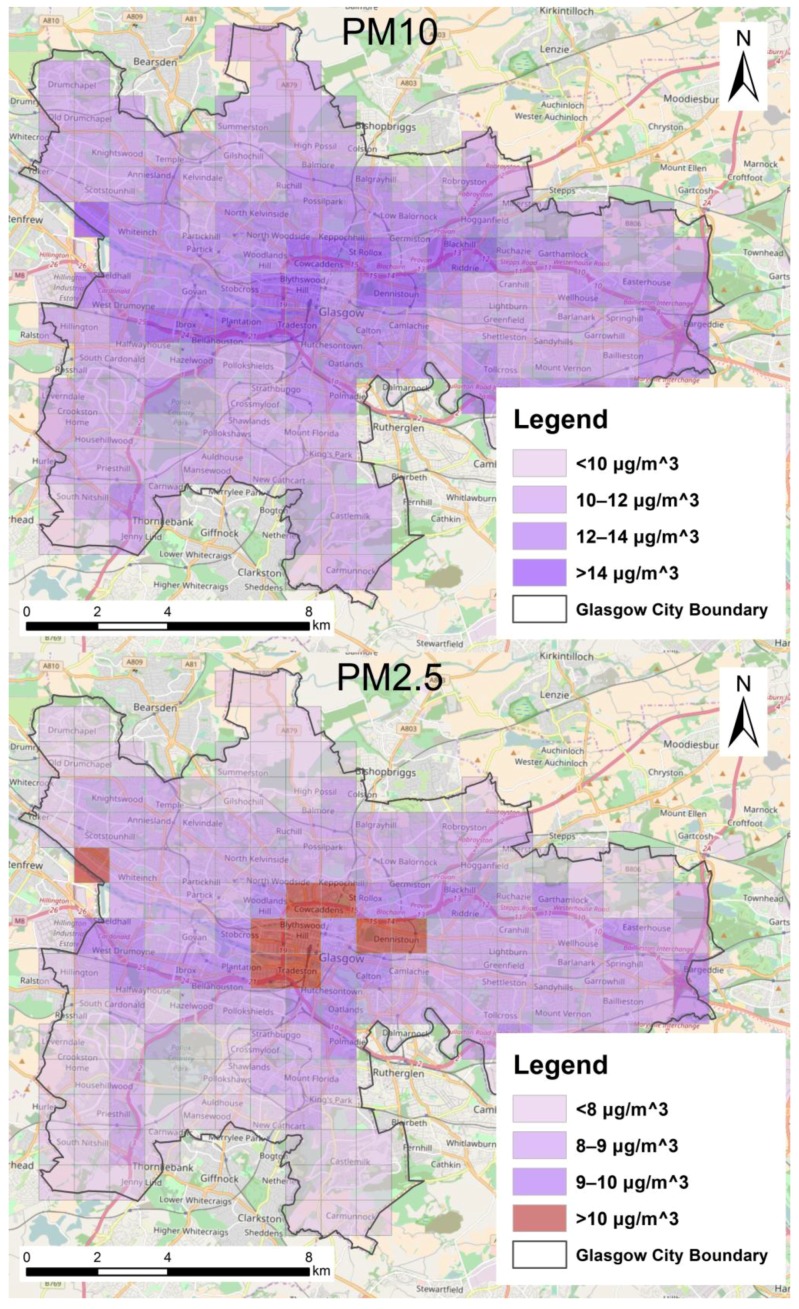
PM grids in Glasgow to represent levels of PM_10_ and PM_2.5_ (source: Scottish Air Quality Database, SAQD).

In this paper, particulate matter (PM), including PM_10_ (coarse PM) and PM_2.5_ (fine PM) are used as the air pollutants to measure levels of air pollution [34,35,36,37,38,39]. PM_10_ is PM with a diameter of 10 micrometers or less; while PM_2.5_ is PM with a diameter of 2.5 micrometers or less. Exposure to air pollution tends to increase risk of disease and mortality [33,44]. For instance, earlier studies provide much evidence that long-term exposure to PM is associated with an increase in cardiovascular and respiratory diseases [33,34,35,36,37,38,39,40] and recently, some studies also reveal short-term exposure to PM is associated with increased mortality risk [41,42,43,44]. According to a report released by WHO [32], Glasgow is one of the worst UK cities for both PM_10_ and PM_2.5_. In this study, background maps for PM_2.5_ and PM_10_ are downloaded from Air Quality in Scotland [53]. The background pollutant concentration maps are presented in 1 km × 1 km grid squares across Scotland. The background maps (reference year 2013) contain estimates of pollutant concentrations (PM_10_ and PM_2.5_) based on an average over a year (annual average) for 2015, 2020, 2025 and 2030 from a base year of 2013 [54]. The 2013 maps are based on ambient monitoring and meteorological data for 2013 [54]. Air pollution background concentration maps for 2015 are used in this study. Specifically, each grid has values to represent annual average estimates of PM_2.5_ and PM_10_ concentrations in 2015 (see Figure 4). The unit of PM_2.5_ and PM_10_ is μg/m^3^. The modelling methodology of PM_10_ background maps is based on Scottish monitoring data and Scottish meteorological data, used to model the annual mean background and roadside concentrations for Scotland; whilst the modelling methodology of PM_2.5_ background maps is based on the UK Pollution Climate Mapping (PCM) approach, used to model the annual mean background and roadside concentrations of PM_2.5_ for the UK as a whole [54].

Only grids that have more than half of their area included in the administrative boundaries of Glasgow are included in the study, resulting in a total of 175 grids. In Glasgow, air pollution levels are observed to have been decreasing in recent years. For instance, the PM_10_ annual mean has decreased from 2010 to 2015 [55]. The main source of air pollution produced is road traffic while the other sources are of less significance [55]. Accordingly, areas with relatively high levels of PM_10_ and PM_2.5_ are in or closed to the city center; while levels of PM_10_ and PM_2.5_ tend to decrease from the city center to outskirts of the city (see Figure 4) where the volume of motor vehicles would be expected to be lower. WHO set 20 μg/m^3^ as a safety guideline for annual mean of PM_10_, and 10 μg/m^3^ as a safety guideline for annual mean of PM_2.5_ [32] and Glasgow is below the safety guideline for PM_10_ for all the areas (20 μg/m^3^). Annual average PM_2.5_ for several areas (grids) that are located in the city center is above the safety guideline (10 μg/m^3^), while annual average PM_2.5_ for the other areas (grids) is below the safety guideline (10 μg/m^3^). We mark areas (grids) with an annual average PM_2.5_ exceeding the safety guideline in red (see Figure 4).

### 2.2. Non-Commuting Cycling Activities and Air Pollution Exposure

#### 2.2.1. Spatial Patterns of Non-Commuting Cycling Activities

Firstly, we define the *non-commuting rate* to measure dominance of non-commuting activities within an area (census output area). Suppose *i* is an area, *non-commuting rate* of *i* is computed as

(1) $$rate\_non\_act\left(i\right)\;=\frac{num\_non\_\;act\left(i\right)}{num\_non\_\;act\left(i\right)+num\_com\_\;act\left(i\right)}$$

(2) $$num\_non\_\;act\left(i\right)\;=\underset{j\in N_{i}}{\sum }\;num\_non\_\;act^{Node}\left(j\right)$$

(3) $$num\_\mathrm{com}\_\;act\left(i\right)\;=\underset{j\in N_{i}}{\sum }\;num\_\mathrm{com}\_\;act^{Node}\left(j\right)$$

where *num_non_act*(*i*) and *num_com_act*(*i*) are the number of non-commuting and commuting cycling activities in the area *i*. *num_non_act ^Node^*(*j*) and *num_com_act ^Node^*(*j*) are the number of non-commuting and commuting activities in the node *j*. *N_i_* is the set of nodes that are located within the area *i*.

In this paper, the improved AMOEBA (A Multidirectional Optimum Ecotope-Based) algorithm developed by [56] is used to identify clusters of high *non-commuting rate*. As a spatially constrained clustering method, this algorithm is applicable to classification of a large number of areas and identification of irregularly shaped clusters. Here we briefly introduce the improved AMOEBA algorithm based on [56]. Essentially, a region or ecotope is a spatially linked group of areas. A region can thus be defined as a spatially contiguous set of areas. The value of the $G_{i}^{*}$ statistic is used to measure the level of clustering of an attribute *x* around an area. Suppose we run AMOEBA on a study region with *N* areas and an attribute *x* with elements *x_i_*, indicating the value of *x* at area *i*. Let us denote this set of areas as *M*, and $\bar{x}$ and *S* as the mean and the standard deviation of the attribute *x* and let *R* be a sub region of *M* with *n* areas. Duque et al. [56] rewrite the formulation of $G_{i}^{*}$ as follows:

(4) $$G_{R}^{*}=\frac{\sum_{i\in R}x_{i}-n\bar{x}}{S\sqrt{\frac{Nn-n^{2}}{N-1}}}\;$$

Basically, $G_{R}^{*}$ depends on the areas that are in the region *R* and the parameters *N*, $\bar{x}$ and *S* that are obtained from the areas in *M*.

Accordingly, a positive (negative) and statistically significant value of $G_{i}^{*}$ statistic indicates the presence of a cluster of high (low) values of attribute *x* around area *i*. Thus, AMOEBA identifies high-valued, or low-valued, ecotopes (regions) by looking for subsets of spatially connected areas with a high absolute value of the $G_{i}^{*}$ statistic. There is only one parameter, i.e., the significance level threshold, that is required to run the AMOEBA algorithm. The significance level threshold was set to 0.01, meaning only clusters with a *p*-value less than 0.01 are statistically significant.

#### 2.2.2. Comparison of Air Pollution Exposure by Cycling Purpose

We quantitatively investigate whether cyclists riding for non-commuting (recreation and other purposes) are more likely to be exposed to lower level of PMs (PM_10_ and PM_2.5_) than cyclists riding for commuting purpose at the node level (the intersection level). First, we compare means of instantaneous exposure to PMs for non-commuting and commuting cycling activities. Second, we compare percentages of ‘high exposure’ activities for non-commuting and commuting cycling activities. As some areas exceed the WHO guideline value for annual PM_2.5_ level (see Figure 4), we call activities that are located within areas (PM grids) of ‘high’ PM_2.5_ levels (annual PM_2.5_ > the WHO guideline: 10 μg/m^3^) ‘high exposure’ activities in this paper.

A more reasonable approach to assess the exposure of cyclists to PM concentrations should take account of not only where cyclists are riding but also the time spent cycling to calculate cyclists’ inhaled dose of PM_10_ or PM_2.5_ air pollution [28]. Ideally, the comparison of air pollution exposure by cycling purpose should be based on cyclists’ inhaled dose of air pollution when riding for commuting and non-commuting. As the Strava Metro do not contain data on time spent cycling, trip distance, or trip speed at the individual level (level of the cyclist), they can’t support assessment of cyclists’ intake of PM_10_ or PM_2.5_ air pollution. Therefore, in this paper, we use instantaneous exposure to PM_10_ or PM_2.5_ air pollution based on only locations of cycling activities.

Suppose there is one cycling activity at a node, meaning that there is one cyclist at this node at a particular time. We could use levels of PM_10_ or PM_2.5_ at a node (street intersection) to represent exposure of the cyclist to PM_10_ or PM_2.5_ air pollution at the moment when he or she is at that node. In other words, levels of PM_10_ or PM_2.5_ at a node where a cycling activity is located could be used to represent instantaneous exposure of the cyclist to PM_10_ or PM_2.5_ air pollution. We could calculate instantaneous exposure of the cyclist to PM_10_ or PM_2.5_ air pollution for each non-commuting or commuting cycling activity. Accordingly, we could calculate means of instantaneous exposure of the cyclist to PM_10_ or PM_2.5_ air pollution for all non-commuting cycling activities and for all commuting cycling activities by

(5) $${\bar{PM}}_{10}^{NON}=\frac{\sum_{i\in S}Num^{NON}\left(i\right)*PM_{10}\left(i\right)}{\sum_{i\in S}Num^{NON}\left(i\right)}\;$$

(6) $${\bar{PM}}_{10}^{COM}=\frac{\sum_{i\in S}Num^{COM}\left(i\right)*PM_{10}\left(i\right)}{\sum_{i\in S}Num^{COM}\left(i\right)}\;$$

(7) $${\bar{PM}}_{2.5}^{NON}=\frac{\sum_{i\in S}Num^{NON}\left(i\right)*PM_{2.5}\left(i\right)}{\sum_{i\in S}Num^{NON}\left(i\right)}\;$$

(8) $${\bar{PM}}_{2.5}^{COM}=\frac{\sum_{i\in S}Num^{COM}\left(i\right)*PM_{2.5}\left(i\right)}{\sum_{i\in S}Num^{COM}\left(i\right)}\;$$

where *i* is a node and *S* is set of nodes. $Num^{NON}\left(i\right)$ and $Num^{COM}\left(i\right)$ are numbers of non-commuting activities and commuting activities in node *i*. $PM_{2.5}\left(i\right)$ and $PM_{10}\left(i\right)$ are the PM_10_ and PM_2.5_ values in node *i*.

Moreover, percentages of ‘high exposure’ activities for non-commuting and commuting cycling activities are calculated by

(9) $$Per_{2.5}^{NON}=\frac{\sum_{j\in H_{2.5}}Num^{NON}\left(j\right)}{\sum_{i\in S}Num^{NON}\left(i\right)}\;$$

(10) $$Per_{2.5}^{COM}=\frac{\sum_{j\in H_{2.5}}Num^{COM}\left(j\right)}{\sum_{i\in S}Num^{COM}\left(i\right)}$$

where *i* is a node and *S* is set of nodes. $Num^{NON}\left(i\right)$ and $Num^{COM}\left(i\right)$ are numbers of non-commuting activities and commuting activities in node *i*. *H*_2.5_ is a sub set of *S*, and it consists of nodes that are located in areas (PM grids) with ‘high’ PM_2.5_ levels (annual PM_2.5_ > the WHO guideline: 10 μg/m^3^). And *j* is a node in sub set *H*_2.5_.

### 2.3. Estimation of Non-Commuting Cycling Activities

In this paper, we try to estimate the number of non-commuting cycling activities at the node level. This means that (1) the dependent variable is the number of non-commuting cycling activities in a node; and (2) the independent variables are the number of all-purpose cycling activities in the node and locational characteristics of the node. Earlier studies investigate impacts of environmental characteristics, including land use mix, cycling facilities, volume or mix of motor vehicle, green space and water, on cycling behavior [57,58,59,60,61]. Table 1 lists the independent variables used in the estimation of non-commuting cycling activities. We select the locational characteristics as the independent variables according to the environmental characteristics from earlier studies and data availability. Specifically, *Dis_to_Greenspace* and *Dis_to_Waterbody* representing distance from node to its nearest rail station and to its nearest water body are used to measure presence or proximity of green space and water. *Dis_to_Citycentre* representing distance from node to the city center is used to reflect levels of land use mix, assuming that the closer to the city center, the higher the level of land use mix is likely to be. *Num_nearest_busstops* representing number of bus stops within a distance of 100 m to node is used to reflect volume or mix of motor vehicles, assuming that the more bus stops there are around, the higher the volume or mix of motor vehicle is likely to be.

In addition to ordinary least squares (OLS), the most widely used model, three other regression models: multilayer perceptron neutral network (MLP), support vector machine (SVM) and random forest (RF) are used to estimate non-commuting cycling activities according to the independent variables when independent variables are not linearly correlated with the dependent variable. We used a cross validation method to evaluate the performances of the four models.

## 3. Results and Discussion

This section demonstrates the empirical results in the study area and makes discussions about the results.

### 3.1. Non-Commuting Cycling Activities and Air Pollution Exposure

#### 3.1.1. Spatial Patterns of Non-Commuting Cycling Activities

In this section, an investigation of spatial patterns of non-commuting cycling activities in Glasgow is demonstrated. In the input of the AMOEBA algorithm, an observation is the *non-commuting rate* of an area (census output area) (see Equations (1)–(3)). As census output area is the area unit, the entire study region (Glasgow) consists of 5486 areas (census output areas) (see Figure 3). In this paper, running AMOEBA is conducted using ClusterPy. ClusterPy is a Python library of spatially constrained clustering algorithms [62].

In the output of AMOEBA, there are ‘solution values’ representing clusters of high values or low values. Specifically, areas with positive ‘solution values’ belong to high value clusters; areas with negative ‘solution values’ belong to low value clusters; and areas with ‘solution values’ of zero are those outside the clusters. Table 2 shows how we group areas with ‘solution values’ to three cluster types: *cluster of high value*, *cluster of low value* and *outside of cluster*. In this empirical study, the *value* here is the *non-commuting rate* of an area.

**Figure 5:**
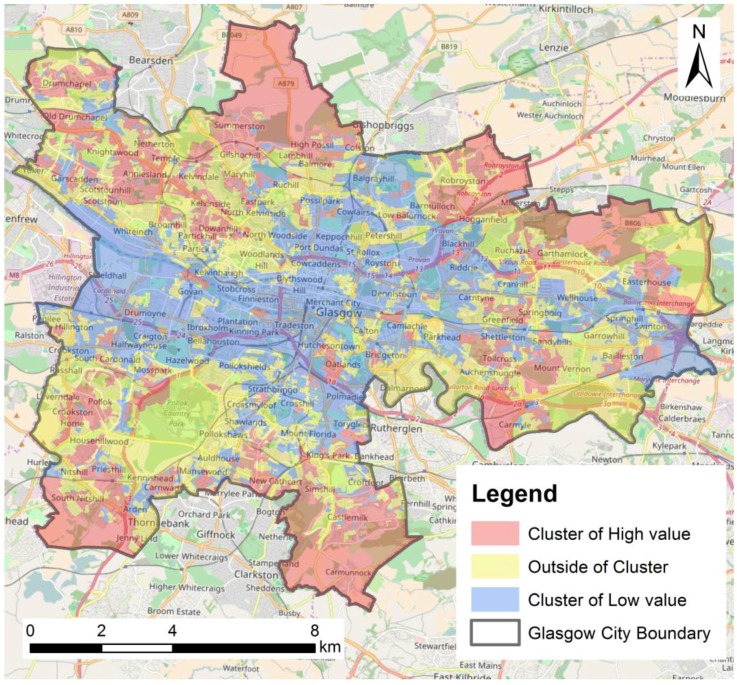
Clusters of high and low *non-commuting rate.*

As a result, we map clusters of high and low value *non-commuting rate* in Glasgow (see Figure 5) which reveals that clusters of high value *non-commuting rate* tend to be located in outskirts of the city, away from the city center. This indicates that non-commuting cycling activities are more likely to be located in outskirts of the city.

#### 3.1.2. Comparison of Air Pollution Exposure by Cycling Purpose

Through viewing Figure 4 and Figure 5 in combination we can infer that non-commuting cycling activities are less likely to be in areas of relatively high levels of PM than commuting cycling activities at the census output area level. Table 3 lists percentages of areas of clusters of high and low *non-commuting rate* with different levels of PM_10_ and PM_2.5_. 80% of clusters of high *non-commuting rate* and only 30% of clusters of low *non-commuting rate* are located within grids with a relatively low PM_10_ level (e.g., below 12 μg/m^3^); whilst 70% of clusters of low *non-commuting rate* and only 20% of clusters of high *non-commuting rate* are located within grids with a relatively high PM_10_ level (e.g., 12 μg/m^3^ and above). Similarly, 96% of clusters of high *non-commuting rate* and only 58% of clusters of low *non-commuting rate* are located within grids with a relatively low PM_2.5_ level (e.g., <9 μg/m^3^); whilst 42% of clusters of low *non-commuting rate* and only 4% of clusters of high *non-commuting rate* are located within grids with a relatively high PM_2.5_ level (e.g., 9 μg/m^3^ and above).

Here we quantitatively investigate whether cyclists riding for recreation and other purposes are more likely to be exposed to lower levels of PMs (PM_10_ and PM_2.5_) than cyclists riding for commuting purpose at the node level (the intersection level). Firstly, the means of instantaneous exposure to PM_10_ and PM_2.5_ for non-commuting and commuting cycling activities are calculated by Equations (5)–(8). Table 4 lists the means of instantaneous exposure to PM_10_ and PM_2.5_ for non-commuting and commuting cycling activities. ${\bar{PM}}_{10}^{NON}$ < ${\bar{PM}}_{10}^{COM}$ and ${\bar{PM}}_{2.5}^{NON}$ < ${\bar{PM}}_{2.5}^{COM}$ indicate that the means of instantaneous exposure to PM_10_ and PM_2.5_ for non-commuting cycling activities are smaller than those for commuting cycling activities. We use the Wilcoxon test to statistically test whether the mean of one group is substantially larger or smaller than the mean of the other group. The Wilcoxon test is used as an alternative to the *T*-test when the data cannot be assumed to be normally distributed. Table 4 also lists results of the Wilcoxon test. In the results of the Wilcoxon test, the *p*-values corresponding to PM_10_ and PM_2.5_ are all less than 0.001. This indicates that the means of instantaneous exposure to PM_10_ and PM_2.5_ for non-commuting cycling activities are both statistically significantly smaller than those for commuting cycling activities at the 0.01 level. This indicates that spatially speaking, cyclists riding for non-commuting purposes tend to be exposed to lower levels of instantaneous PM_10_ and PM_2.5_ air pollution than cyclists riding for commuting purposes.

Moreover, we calculate and compare percentages of ‘high exposure’ activities for non-commuting and commuting cycling activities by Equations (9) and (10). Table 5 lists percentages of ‘high exposure’ activities for non-commuting and commuting cycling activities. $Per_{2.5}^{COM}$ is two times larger than $Per_{2.5}^{NON}$, indicating that cyclists riding for commuting purposes are more likely to pass through areas of ‘high’ PM_2.5_ levels than cyclists riding for non-commuting purposes.

In summary, the empirical results reveal that: spatially speaking, cyclists riding for recreation and other purposes are more likely to be exposed to lower level of PMs than cyclists riding for commuting purposes. The means of instantaneous exposure to PM_10_ and PM_2.5_ for non-commuting cycling activities are smaller than those for commuting cycling activities. In addition to encouraging commuters to ride bikes on working days, encouraging people to ride bikes for recreation on non-working days and holidays may contribute to development of urban sustainability. As recreational cyclists are more likely to be in outskirts of cities, policymakers might consider how to improve cycling infrastructure and road safety in those areas when designing or changing urban infrastructure.

### 3.2. Estimation of Non-Commuting Cycling Activities

**Figure 6:**
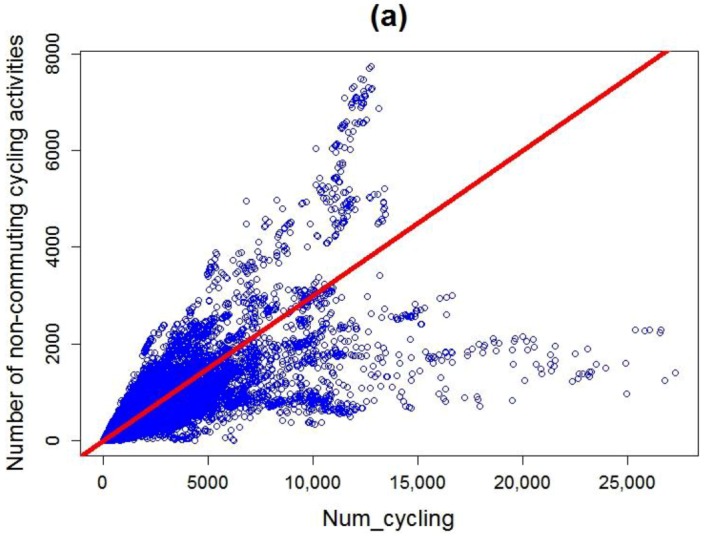

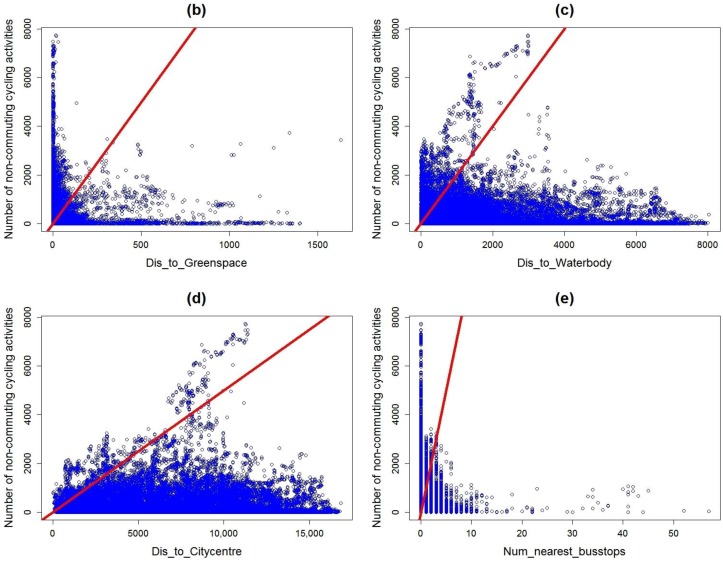
Scatterplots generated for each independent variable with the dependent variable: (**a**) Num_cycling vs. the dependent variable; (**b**) Dis_to_Greenspace vs. the dependent variable; (**c**) Dis_to_Waterbody vs. the dependent variable; (**d**) Dis_to_Citycentre vs. the dependent variable; (**e**) Num_nearest_busstops vs. the dependent variable.

The 50,057 nodes used in the spatial analysis constitute the data set used in the estimation of non-commuting cycling activities as well. We further calculate the locational characteristics for each node (see Table 1 in Section 2). An explorative analysis is made to know whether each independent variable is linearly correlated with the dependent variable. Figure 6 shows the scatterplots generated for each independent variable with the dependent variable. Apart from *Num_cycling*, other independent variables do not have a significant correlation with the dependent variable as absolute values of the corresponding Pearson correlation coefficients are less than 0.1. Therefore, in addition to OLS, three other models are also used to estimate non-commuting cycling activities.

**Figure 7:**
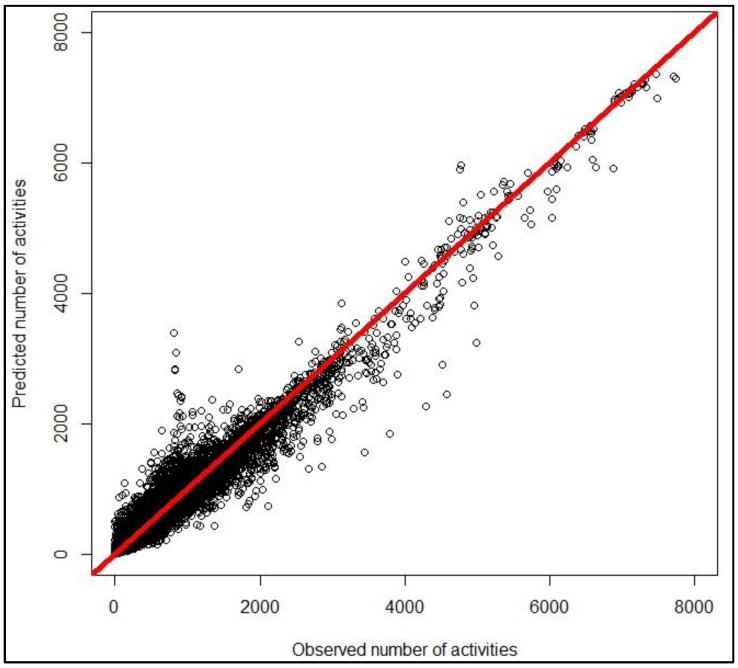
Predicted and observed number of non-commuting activities.

We run a 10-folder cross validation to measure the performances of the four estimation models. Correlation of predicted and actual number of non-commuting activities is used to measure estimation performance. Random forest (RF) outperforms other algorithms with a correlation coefficient of 0.981 (see Table 6). Figure 7 plots the predicted and actual number of non-commuting activities. The estimation results indicate that the estimation of the number of non-commuting cycling activities is fairly good in this study. This suggests that we may be able to estimate the number of non-commuting cycling trips when the trip purpose of cycling data is unknown.

## 4. Conclusions

In this study, we investigate spatial patterns of cycling activities and associations between cycling purpose and air pollution exposure in Glasgow, UK by using Strava Metro data. Empirical results reveal some findings that (1) compared with commuting cycling activities, non-commuting cycling activities are more likely to be located in outskirts of the city; (2) spatially speaking, cyclists riding for recreation and other purposes are more likely to be exposed to relatively low levels of air pollution than cyclists riding for commuting; and (3) the method for estimating of the number of non-commuting cycling activities works well in this study. The results suggest that (1) policymakers might consider how to improve cycling infrastructure and road safety in outskirts of cities; and (2) we may be able to estimate the number of non-commuting cycling activities when trip purpose of cycling data is unknown. We conclude that this study is a good start in utility of crowdsourced cycling data for studies of cycling and air pollution exposure.

### 4.1. Limitations

This paper does present a few limitations. First, a census output area is used as the area unit in in the identifying clusters. The modifiable areal unit problem (MAUP) might influence the cluster identification in this study. Second, although the estimation of non-commuting cycling activities is good at the node level, the estimation at the edge (street) level is unknown. Third, although the models work well in estimating non-commuting cycling activities, there might be some potential to improve the estimation. Ideally, we could improve the estimation by incorporating more attributes such as land use mix, residential density, traffic count, road type, road width, etc., into the models. We are not able to include those attributes due to present data availability. Fourth, instantaneous assessment of air pollution is used in this study. In fact, cumulative assessment of air pollution makes more sense to studies of health effects of active travel. Ideally, the inhaled dose of air pollution during a cycling trip should be assessed according to not only where the trip takes place but also the time spent travelling. Furthermore, long-term air pollution exposure of a cyclist should also take account of the number of his or her commuting trips and non-commuting trips within a longer period (e.g., one year or more). As Strava Metro doesn’t offer individual-level trips, we know neither how long a commuting or non-commuting cycling trip takes nor how many commuting or non-commuting cycling trips each biker takes in one year. Thus, we are not able to assess cumulative exposure of a cyclist to air pollution. Finally, since VGI, UGC and crowdsourced data are collected and shared by individuals, there are arguments about the quality and fitness for use of such data in projects [63]. While we are aware of this issue, we do not tackle this in this study, as it requires separate study on this topic.

### 4.2. Future Works

In the future, we will take account of some aspects to enhance this study. First, as the Strava database offered by Urban Big Data Centre offers the number of cycling activities in distinct daily time slots, we could model spatio-temporal variations in non-commuting cycling activities according to spatio-temporal characteristics; Second, we will incorporate other air pollutants such as sulphur dioxide, nitrogen dioxide and ozone into the analysis. In addition, although Strava Metro doesn’t offer individual-level trips, we may be able to assess cumulative air pollution exposure by using the sub data set ‘Streets’ in Strava Metro. Based on the number of trips on each street segment, we could use length of the street segment to represent the length of sub cycling trip, and then estimate the time of sub cycling trip based on average speed of cycling for commuting or recreation and other purposes in Glasgow, a figure we could possibly obtain from Strava Metro or other data sources (e.g., travel surveys). Accordingly, we could estimate the total air pollution exposure to all of Glasgow’s Strava cyclists when riding for commuting or recreation and other purposes during one year, and further estimate annual average air pollution exposure of one cyclist when riding for commuting or recreation and other purposes. This would represent a large amount of future work and potential to gain a much greater understanding of the impact of city air pollution.

## Figures and Tables

**Table 1:** Independent variables in the estimation of non-commuting cycling activities.

Variable	Meaning
Num_cycling	Number of cycling activities at the node
Dis_to_Greenspace	Distance from node to its nearest rail station
Dis_to_Waterbody	Distance from node to its nearest water body
Dis_to_Citycentre	Distance from node to the city center
Num_nearest_busstops	Number of near bus stops (number of bus stops within a distance of 100 m to node)

**Table 2:** Cluster types and associated solution values.

Solution Value	Cluster Type
≥1	Cluster of High value
0	Outside of Cluster
≤−1	Cluster of Low value

**Table 3:** Percentages of areas of clusters of high and low *non-commuting rate* with different levels of PM_10_ and PM_2.5_.

Percent of Areas		Clusters of High and Low *Non-Commuting Rate*		
		Cluster of High Value	Outside of Cluster	Cluster of Low Value
PM_10_ (Unit: μg/m^3^)	<0	0%	0%	0%
	10–12	80%	61%	30%
	12–14	19%	36%	46%
	>14	1%	3%	24%
PM_2.5_ (Unit: μg/m^3^)	<8	60%	33%	8%
	8–9	36%	58%	50%
	9–10	4%	8%	28%
	>10	0%	1%	14%

**Table 4:** Means of instantaneous exposure to PM_10_ and PM_2.5_ for non-commuting and commuting cycling activities.

Air Pollution Exposure	${\bar{PM}}_{10}^{NON}$	${\bar{PM}}_{10}^{COM}$	${\bar{PM}}_{2.5}^{NON}$	${\bar{PM}}_{2.5}^{COM}$
Unit: μg/m^3^	11.823	12.631	8.233	8.753
Wilcoxo*n* Test *p-*value	<0.001		<0.001	

**Table 5:** Percentages of ‘high exposure’ activities for non-commuting and commuting cycling activities.

$Per_{2.5}^{NON}$	$Per_{2.5}^{COM}$
6.4%	15.0%

**Table 6:** Estimation results of non-commuting cycling activities by different algorithms.

Accuracy	OLS	MLP	SVM	RF
Correlation coefficient (Pearson’s *R*)	0.814	0.904	0.807	0.981

OLS: ordinary least squares; MLP: multilayer perceptron neutral newtork; SVM: support vector machine; RF: random forest.

## References

[1] Cavill N., Davis A. (2007). Cycling and Health: What’s the Evidence?.

[2] Forsyth A., Krizek K.J., Agrawal A.W., Stonebraker E. (2012). Reliability testing of the Pedestrian and Bicycling Survey (PABS) method. J. Phys. Act. Health.

[3] Oja P., Vuori I., Paronen O. (1998). Daily walking and cycling to work: Their utility as health-enhancing physical activity. Patient Educ. Couns..

[4] Oja P., Titze S., Bauman A., de Geus B., Krenn P., Reger-Nash B., Kohlberger T. (2011). Health benefits of cycling: A systematic review. Scand. J. Med. Sci. Sports.

[5] Pucher J., Buehler R., Bassett D.R., Dannenberg A.L. (2010). Walking and cycling to health: A comparative analysis of city, state, and international data. Am. J. Public Health.

[6] Taddei C., Gnesotto R., Forni S., Bonaccorsi G., Vannucci A., Garofalo G. (2015). Cycling Promotion and Non-Communicable Disease Prevention: Health Impact Assessment and Economic Evaluation of Cycling to Work or School in Florence. PLoS ONE.

[7] Wen L.M., Rissel C. (2008). Inverse associations between cycling to work, public transport, and overweight and obesity: Findings from a population based study in Australia. Prev. Med..

[8] Elwood S., Goodchild M.F., Sui D.Z. (2012). Researching volunteered geographic information: Spatial data, geographic research, and new social practice. Assoc. Am. Geogr..

[9] Griffin G.P., Jiao J. (2015). Where does bicycling for health happen? Analysing volunteered geographic information through place and plexus. J. Transp. Health.

[10] Forsyth A., Oakes J.M. (2015). Cycling, the Built Environment, and Health: Results of a Midwestern Study. Int. J. Sustain. Transp..

[11] Sener I.N., Eluru N., Bhat C.R. (2009). An analysis of bicycle route choice preferences in Texas, US. Transportation.

[12] El Esawey M. (2014). Estimation of annual average daily bicycle traffic with adjustment factors. Transp. Res. Rec..

[13] Jesticoa B., Nelsona T., Wintersb M. (2016). Mapping ridership using crowdsourced cycling data. J. Transp. Geogr..

[14] Broach J., Dill J., Gliebe J. (2012). Where do cyclists ride? A route choice model developed with revealed preference GPS data. Transp. Res. Part A Policy Pract..

[15] Casello J.M., Usyukov V. (2014). Modeling cyclists’ route choice based on GPS data. Transp. Res. Rec..

[16] Hood J., Sall E., Charlton B. (2011). A GPS-based bicycle route choice model for San Francisco, California. Transp. Lett..

[17] Heesch K.C., James B., Washington T.L., Zunig K., Burke M. (2016). Evaluation of the Veloway 1: A natural experiment of new bicycle infrastructure in Brisbane, Australia. J. Transp. Health.

[18] Strava Metro (2016). Data-Driven Bicycle and Pedestrian Planning. http://metro.strava.com/.

[19] Bascom R., Bromberg P.A., Costa D.L., Devlin R., Dockery D.W., Frampton M.W., Lambert W., Samet J.M., Speizer F.E., Utell M. (1996). Health effects of outdoor air pollution. Am. J. Respir. Crit. Care Med..

[20] Cao J., Yang C., Li J., Chen R., Chen B., Gu D., Kan H. (2011). Association between long-term exposure to outdoor air pollution and mortality in China: A cohort study. J. Hazard. Mater..

[21] Künzli N., Kaiser R., Medina S., Studnicka M., Chanel O., Filliger P., Herry M., Horak F., Puybonnieux-Texier V., Quénel P. (2000). Public-health impact of outdoor and traffic-related air pollution: A European assessment. Lancet.

[22] Briggs D.J., de Hoogh K., Morris C., Gulliver J. (2008). Effects of travel mode on exposures to particulate air pollution. Environ. Int..

[23] Chertok M., Voukelatos A., Sheppeard V., Rissel C. (2004). Comparison of air pollution exposure for five commuting modes in Sydney—Car, train, bus, bicycle and walking. Health Promot. J. Aust..

[24] De Nazelle A., Fruin S., Westerdahl D., Martinez D., Ripoll A., Kubesch N., Nieuwenhuijsen M. (2012). A travel mode comparison of commuters’ exposures to air pollutants in Barcelona. Atmos. Environ..

[25] Gulliver J., Briggs D. (2005). Time-space modeling of journey-time exposure to traffic-related air pollution using GIS. Environ. Res..

[26] Rojas-Rueda D., de Nazelle A., Tainio M., Nieuwenhuijsen M.J. (2011). The health risks and benefits of cycling in urban environments compared with car use: Health impact assessment study. BMJ.

[27] Zuurbier M., Hoek G., Oldenwening M., Lenters V., Meliefste K., van den Hazel P., Brunekreef B. (2010). Commuters’ exposure to particulate matter air pollution is affected by mode of transport, fuel type, and route. Environ. Health Perspect..

[28] Tainio M., de Nazelle A.J., Götschi T., Kahlmeier S., Rojas-Reuda D., Nieuwenhuijsen M.J., De sa Herick T., Kelly P., Woodcock J. (2016). Can air pollution negate the health benefits of cycling and walking?. Prev. Med..

[29] De Nazelle A., Seto E., Donaire-Gonzalez D., Mendez M., Matamala J., Nieuwenhuijsen M.J., Jerrett M. (2013). Improving estimates of air pollution exposure through ubiquitous sensing technologies. Environ. Pollut..

[30] Weichenthal S., Kulka R., Dubeau A., Martin C., Wang D., Dales R. (2011). Traffic-related air pollution and acute changes in heart rate variability and respiratory function in urban cyclists. Environ. Health Perspect..

[31] Hollingworth M., Harper A., Hamer M. (2014). An Observational Study of Erectile Dysfunction, Infertility, and Prostate Cancer in Regular Cyclists: Cycling for Health UK Study. J. Men’s Health.

[32] WHO (2016). Air Pollution Levels Rising in Many of the World’s Poorest Cities. http://www.who.int/mediacentre/news/releases/2016/air-pollution-rising/.

[33] Anderson J.O., Thundiyil J.G., Stolbach A. (2012). Clearing the Air: A Review of the Effects of Particulate Matter Air Pollution on Human Health. J. Med. Toxicol..

[34] Dockery D.W., Pope C.A., Xu X., Spengler J.D., Ware J.H., Fay M.E., Ferris B.G., Speizer F.E. (1993). An association between air pollution and mortality in six U.S. cities. N. Engl. J. Med..

[35] Dominici F., Peng R.D., Bell M.L., Pham L., McDermott A., Zeger S.L., Samet J.M. (2006). Fine particulate air pollution and hospital admission for cardiovascular and respiratory diseases. JAMA.

[36] Hoek G., Brunekreef B., Goldbohm S., Fischer P., van den Brandt P.A. (2002). Association between mortality and indicators of traffic-related air pollution in the Netherlands: A cohort study. Lancet.

[37] Miller K.A., Siscovick D.S., Sheppard L., Shepherd K., Sullivan J.H., Anderson G.L., Kaufman J.D. (2007). Long-term exposure to air pollution and incidence of cardiovascular events in women. N. Engl. J. Med..

[38] Lipsett M.J., Ostro B.D., Reynolds P., Goldberg D., Hertz A., Jerrett M., Smith D.F., Garcia C., Chang E.T., Bernstein L. (2011). Long-Term Exposure to Air Pollution and Cardiorespiratory Disease in the California Teachers Study Cohort. Am. J. Respir. Crit. Care Med..

[39] Pope C.A., Burnett R.T., Thurston G.D., Thun M.J., Calle E.E., Krewski D., Godleski J.J. (2004). Cardiovascular mortality and long-term exposure to particulate air pollution: Epidemiological evidence of general pathophysiological pathways of disease. Circulation.

[40] Pope C.A., Turner M.C., Burnett R.T., Jerrett M., Gapstur S.M., Diver W.R., Krewski D., Brook R.D. (2015). Relationships between fine particulate air pollution, cardiometabolic disorders and cardiovascular mortality. Circ. Res..

[41] Bell M.L., Ebisu K., Peng R.D., Walker J., Samet J.M., Zeger S.L., Dominici F. (2008). Seasonal and regional short-term effects of fine particles on hospital admissions in 202 US counties, 1999–2005. Am. J. Epidemiol..

[42] Chen R., Kan H., Chen B., Huang W., Bai Z., Song G., Pan G. (2012). Association of particulate air pollution with daily mortality: The China Air Pollution and Health Effects Study. Am. J. Epidemiol..

[43] Li M.H., Fan L.C., Mao B., Yang J.W., Choi A.M., Cao W.J., Xu J.F. (2016). Short-Term Exposure to Ambient Fine Particulate Matter Increases Hospitalizations and Mortality in COPD: A Systematic Review and Meta-Analysis. Chest.

[44] Shah A.S., Lee K.K., McAllister D.A., Hunter A., Nair H., Whiteley W., Langrish J.P., Newby D.E., Mills N.L. (2015). Short term exposure to air pollution and stroke: Systematic review and meta-analysis. BMJ.

[45] Doorley R., Pakrashi V., Ghosh B. (2015). Quantifying the health impacts of active travel: Assessment of methodologies. Transp. Rev..

[46] Mueller N., Rojas-Rueda D., Cole-Hunter T., de Nazelle A., Dons E., Gerike R., Götschi T., Int Panis L., Kahlmeier S., Nieuwenhuijsen M. (2015). Health impact assessment of active transportation: A systematic review. Prev. Med..

[47] Lippmann M. (2013). Exposure science in the 21st century: A vision and a strategy. J. Expo. Sci. Environ. Epidemiol..

[48] Riordan B. (2016). Strava Metro: Better Data for Better Cities. http://ubdc.ac.uk/media/1416/uofg-training.pdf.

[49] Strava Metro (2015). Strava Metro Comprehensive User Guide Version 2.0. http://ubdc.ac.uk/media/1323/stravametro_200_user_guide_withoutpics.pdf.

[50] Herrero J. (2016). Using Big Data to Understand Trail Use: Three Strava Tools. https://www.trafx.net/insights.htm.

[51] Urban Big Data Centre UK. Data Services: Strava Metro Data. http://ubdc.ac.uk/data-services/data-catalogue/transport-data.

[52] DATA.GOV.UK Datasets: Output Areas. https://data.gov.uk/dataset.

[53] Ricardo Energy & Environment 2015 Air Quality in Scotland. http://www.scottishairquality.co.uk.

[54] UK Department for Environment Food & Rural Affairs (2016). Background Concentration Maps User Guide. http://laqm.defra.gov.uk.

[55] Ricardo Energy and Environment (2016). Technical Reports—Air Quality Scotland Brochure 2015. http://www.scottishairquality.co.uk.

[56] Duque J.C., Aldstadt J., Velasquez E., Franco J.L., Betancourt A. (2011). A computationally efficient method for delineating irregularly shaped spatial clusters. J. Geogr. Syst..

[57] Carver A., Salmon J., Campbell K., Baur L., Garnett S., Crawford D. (2005). How do perceptions of local neighborhood relate to adolescents’ walking and cycling?. Am. J. Health Promot..

[58] Hunt J.D., Abraham J.E. (2007). Influences on bicycle use. Transportation.

[59] De Vries S.I., Hopman-Rock M., Bakker I., Hirasing R.A., van Mechelen W. (2010). Built environmental correlates of walking and cycling in Dutch urban children: Results from the SPACE study. Int. J. Environ. Res. Public Health.

[60] Fraser S., Lock K. (2010). Cycling for transport and public health: A systematic review of the effect of the environment on cycling. Eur. J. Public Health.

[61] Mäki-Opas T.E., Borodulin K., Valkeinen H., Stenholm S., Kunst A.E., Abel T., Härkänen T., Kopperoinen L., Itkonen P., Prättälä R. (2016). The contribution of travel-related urban zones, cycling and pedestrian networks and green space to commuting physical activity among adults—A cross-sectional population-based study using geographical information systems. BMC Public Health.

[62] RiSE Group ClusterPy: Library of Spatially Constrained Clustering Algorithms. www.rise-group.org/risem/clusterpy.

[63] Senaratne H., Mobasheri A., Ali A.L., Capineri C., Haklay M. (2017). A review of volunteered geographic information quality assessment methods. Int. J. Geogr. Inf. Sci..

